# Unexpected friction behaviours due to capillary and adhesion effects

**DOI:** 10.1038/s41598-017-00238-0

**Published:** 2017-03-10

**Authors:** Fei Guo, Yu Tian, Ying Liu, Yuming Wang

**Affiliations:** State Key Laboratory of Tribology, Tsinghua University, Beijing, 100084 China

## Abstract

In this present work, we show that pouring water into the interface of SiC sliding against the same SiC material makes the friction coefficient far exceed that governing initial dry friction, and a subsequent load increase causes a dramatic abnormal friction force decrease. With the load further increasing to a critical value, friction force sharply increases again. The small amounts of water in the interface generates a large capillary force, the capillary bridges break to cause micro-separation of interface increase to allow bulk water to enter the friction interface, and a squeezing out of the water film to form a strong adhesion force between SiC-SiC materials, are considered to be responsible for the three stages, respectively.

## Introduction

Traditional tribology theory states that, when adding fluid such as oil or water into a dry friction interface, the friction force will usually decrease greatly due to these fluids acting as a lubricant independent of lubrication state; when the external load increases, the friction coefficient might decrease but the friction force would increase^1, 2^. Since people started to understand friction phenomena, these conclusions have been widely used in industry to date; however, abnormal phenomena, not according with traditional theory, always arouse the greatest interest among scientists, which is helpful to promote scientific and technological progress and continuously perfect traditional theories.

Compared to dry friction, the presence of small amounts of water or humid air in the sliding interface can lead to an increase in friction force^3–7^. The general consensus is that small amounts of water can form capillary bridges in a three-phase solid-water-air system. The water bridges can generate a large Laplace pressure, causing a strong internal attraction in the friction interface, which is equivalent to an increase in external load. Not only that, some special materials’ friction interface can even achieve superlubricity (a friction coefficient of less than 0.01) in a dry environment, but when there is only small amounts of water in the friction interface, the friction coefficient will increase to a significant extent^8–11^. More surprisingly, the friction coefficient of a super-hydrophilic poly(MPC) brush sliding against a glass ball probe in water (*i.e*., when the contact interface was immersed in water) was larger than that in normal ambient condition^12^, which was also found for an aluminium-bronze coating sliding against silicon nitride^13^. However, these authors did not propose an effective mechanism by which to interpret this unique frictional property.

In any lubrication state, friction force usually increases with increasing normal load, either linearly or non-linearly, which has been known for centuries from the earliest studies of Leonardo da Vinci and Amontons. Recently, the adhesion-dependent negative friction coefficient on chemically modified graphite surface during retraction of an AFM tip has been measured: the friction force increases with decreasing load so that the slope of the friction force against load curve is negative^14^. The phenomenon was explained by a model where the adhesion between the AFM tip and the graphite surface is stronger than the dispersion forces holding the layers in the graphite together. Subsequently, similar phenomena were also found in other friction interfaces in AFM experiments, between layers of methylcellulose anchored by hydrophobic interactions to a hydrophobised silica surface and a hydrophobised silica micro-particle, and between silica surfaces coated with a hydrophobic, cationic diblock copolymer by means of electrostatic anchoring^15–18^. So, the abnormal law was thought to be a general phenomenon related to hysteresis in the adhesive interaction between two sliding bodies. However, the abnormal finding was only observed at nanoscale between the AFM tip and underlying substrate; to date, there are no published examples confirming this at the macroscale.

In this present work, we utilized the pressureless sintered silicon carbide (SiC) sliding against itself, namely the moving and static rings materials were the same, to demonstrate some unexpected friction behaviours with a macroscopic standard tribometer. At the initial stage, the SiC-SiC friction interface ran under dry sliding conditions, after a period of time, adding water made the friction coefficient increase sharply. Then we increased the external load under water lubrication conditions, finding the anomaly by which the friction force decreased with increasing external load within certain ranges; when the external load continued to increase to a critical value, the friction force dramatically increased so that exceeding the maximum drive torque of the tribometer motor, resulting in the rotator being brought to as enforced stop.

## Results

### Friction behaviours

**Figure 1:**
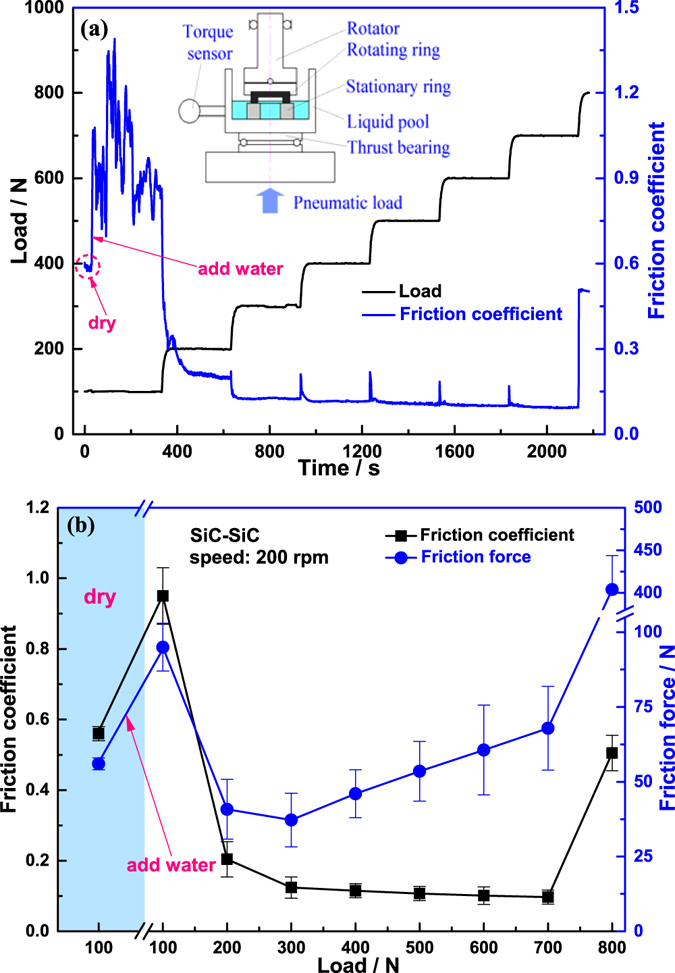
(**a**) Variation of friction coefficient between SiC-SiC friction interface with running time under different external loads at a rate of rotation of 200 rpm, corresponding to 0.5 m/s, from initial dry sliding to the addition of water with a standard Plint TE92 tribometer, the schematic of which is shown in the embedded figure. (**b**) The average value of the above variation curve at each lubrication state, or each load, is taken as the friction coefficient. The tests are repeated three times to check for reproducibility and the average value of the three experiments is taken as the final friction coefficient (the standard deviation is shown as the error bar). The friction force is obtained from the product of the friction coefficient and the external load.

Figure 1 shows the variation of friction coefficient and friction force between SiC-SiC friction interface from the initial dry friction to water lubrication. At the initial dry sliding stage, the friction coefficient stabilized at approximately 0.58 under a load of 100 N; but when adding water into the liquid pool, both the friction coefficient and the curve fluctuation extent dramatically increased immediately, accompanying by a slight vibration of the liquid pool and an obvious reduction in friction noise, which indicated that addition of water did not play a lubricant role but deteriorated the interface friction behaviour. As the load increased to 200 N~300 N, not only did the friction coefficient decrease rapidly, but also the friction force decreased abnormally. Meanwhile, the large extent of the fluctuations in the friction coefficient *versus* time curve decreased and the vibration of the liquid pool, and the friction noise, almost completely disappeared. All of these phenomena indicated that bulk water entered the friction interface and played a lubricating role. As the load further increased within the range from 300 N to 700 N, the friction force underwent a quasi-linear increase, in accordance with Amontons’ law, however, once the load exceeded 700 N, the friction force sharply increased immediately, even causing rotation speed of the tribometer to decrease enforcedly; when the load reached 800 N, the rotator stopped completely, so it is deemed that the range from 700 N to 800 N is a critical load zone, in which SiC-SiC friction interface was unable to run normally.

### Characterization of surface topography and wear debris

**Figure 2:**
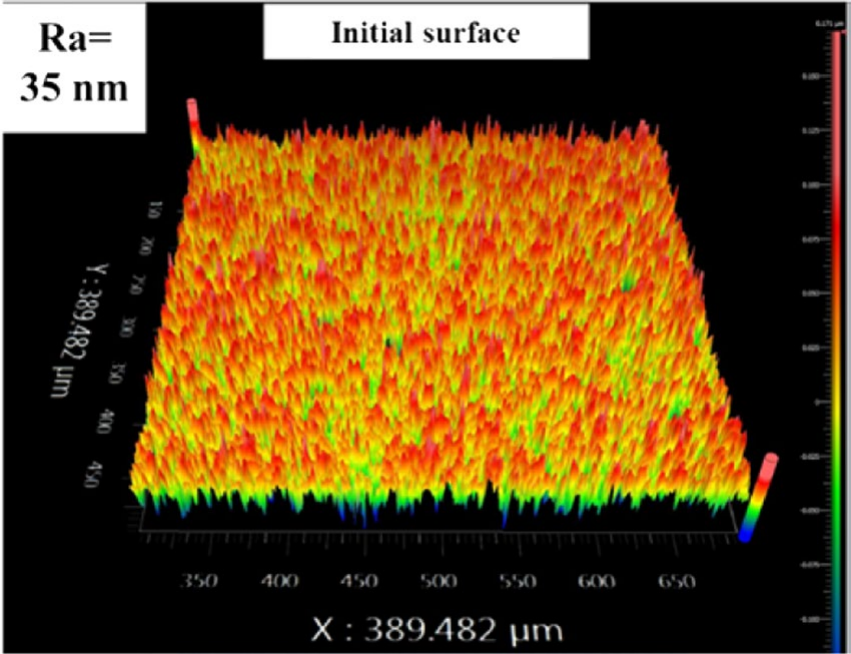
Initial surface topography: The surface asperities distribution is uniform and the surface roughness is 35 nm after grinding using a diamond slurry on a brass grading plate.

**Figure 3:**
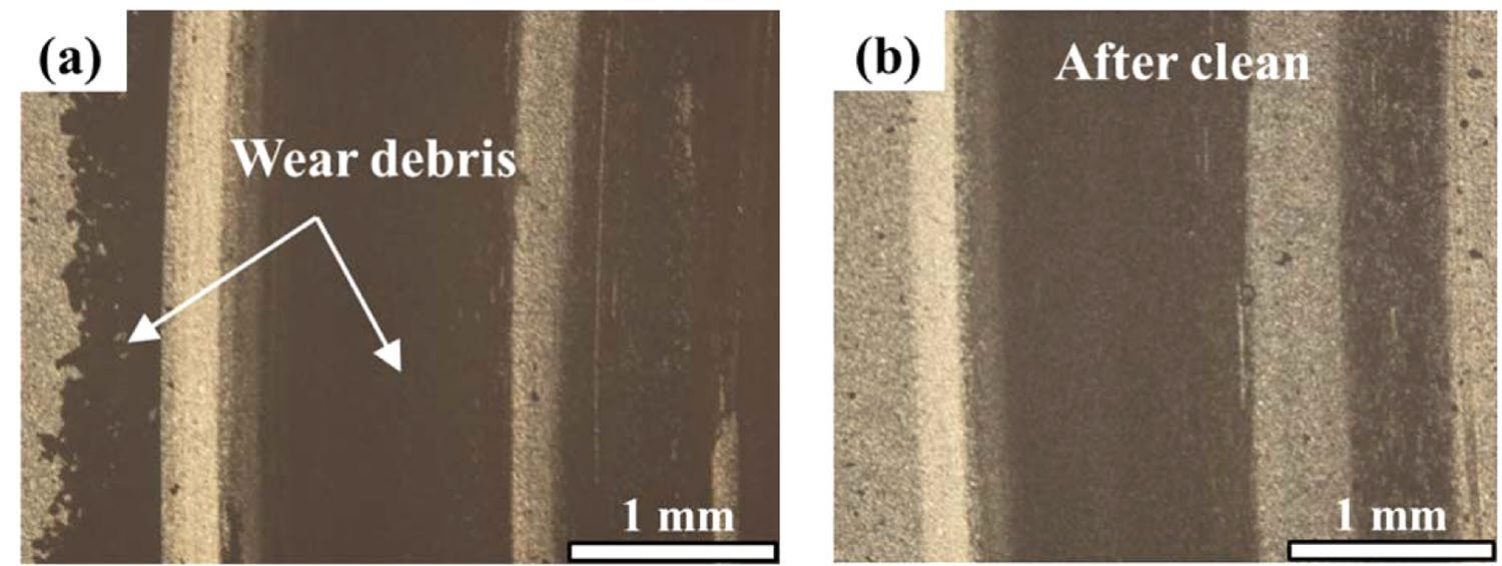
Wear debris on surface (**a**) before and (**b**) after cleaning after initial dry friction under the optical microscope.

**Figure 4:**
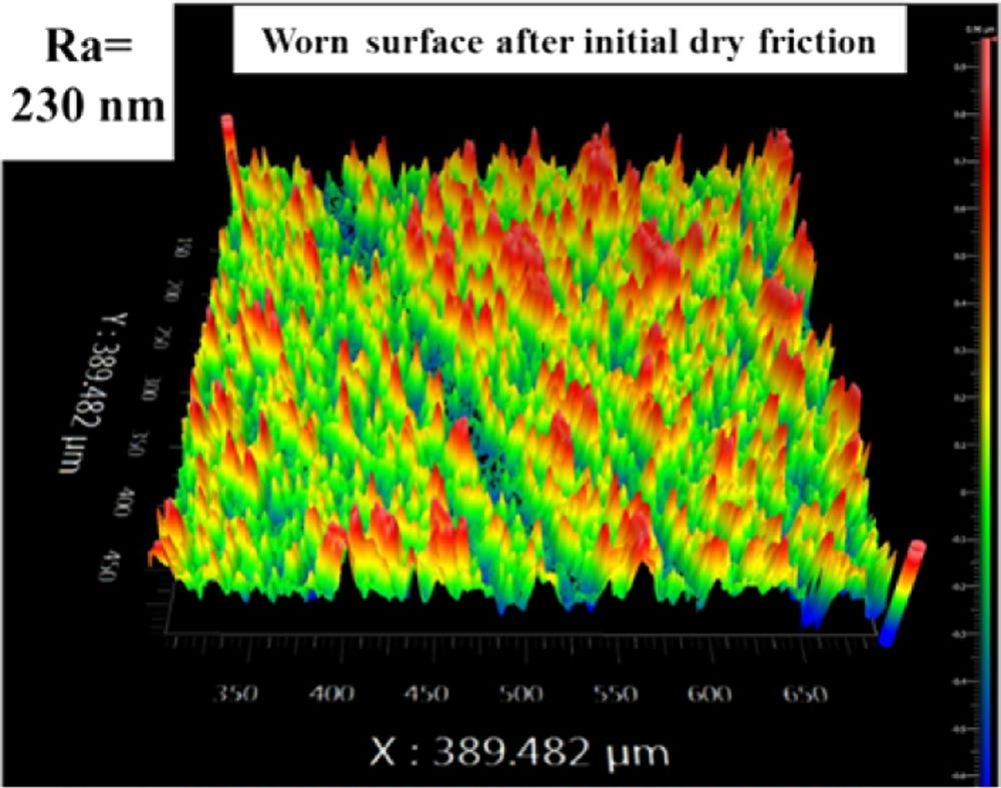
Worn surface topography after ultrasonic cleaning after initial dry friction: The surface is more coarse, and the peak of asperities obviously increases; the surface roughness is 230 nm.

Charactering the surface microtopography and wear debris state is beneficial to understand the friction behaviours between SiC-SiC interface. Figure 2 shows the initial SiC surface topography, and the surface roughness was 35 nm after grinding. After the initial dry friction, there was a lot of obvious wear debris on the SiC surface shown in Fig. 3(a), comparing with the cleaning surface shown in Fig. 3(b). After the ultrasonic cleaning, the worn surface microtopography is shown in Fig. 4, it is seen that the surface is more coarse, and the peak of asperities obviously increases; and the surface roughness is 230 nm.

## Discussion

### Capillary effect

**Figure 5:**
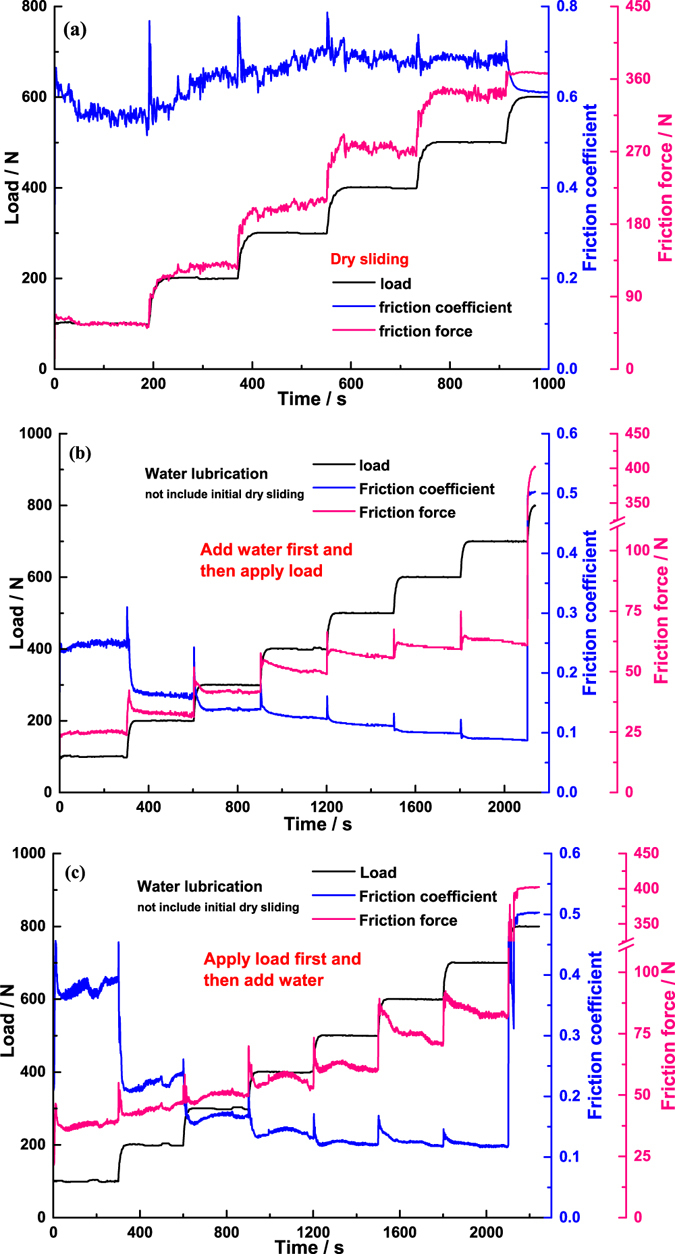
Variation of friction coefficient and friction force between SiC-SiC with running time under different external loads at a rate of rotation of 200 rpm, corresponding to 0.5 m/s, under different lubrication states: (**a**) dry friction, (**b**) water lubrication not including initial dry sliding, and according to the sequence of adding water first and then applying load, and (**c**) water lubrication not including initial dry sliding, and according to the sequence of applying load first then adding water.

**Figure 6:**
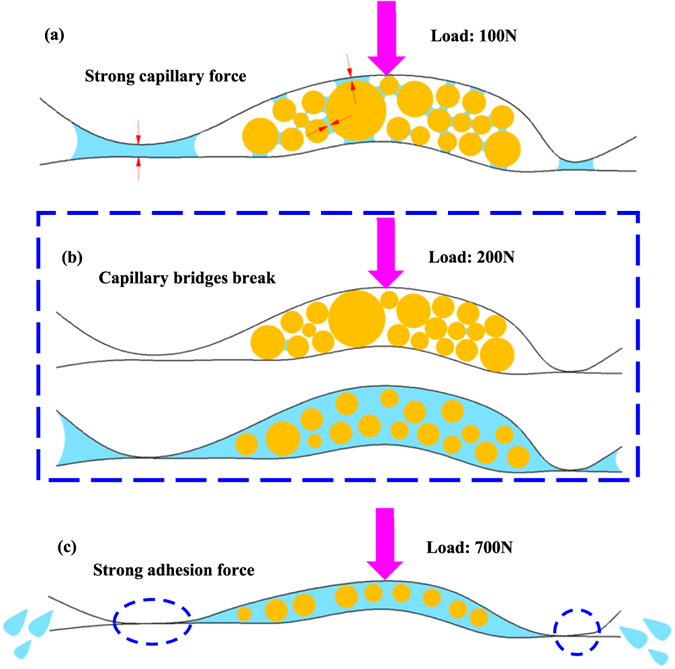
Schematic of the influence mechanism of capillary force and adhesion force in the SiC-SiC friction interface: (**a**) Bulk water does not enter the initial dry friction interface, there is only small amounts of water therein, which forms enormous capillary pressure, causing a strong attraction between asperities, between wear debris, and between asperities and wear debris. (**b**) The increased external load causes the small amounts of water within the three parts to be squeezed out from the capillary bridges to become free water, leading to the equivalent external load suddenly being released, thus resulting in the micro-separation of the interface suddenly increasing, and then generating the passage through which bulk water enters the interface. (**c**) When the load increases to a certain value, exceeding the maximum carrying capacity of the water film, the water in the interface is squeezed out, leading to solid-solid contact area and the contact pressure then increases, thus forming a strong adhesion force between SiC-SiC.

**Figure 7:**
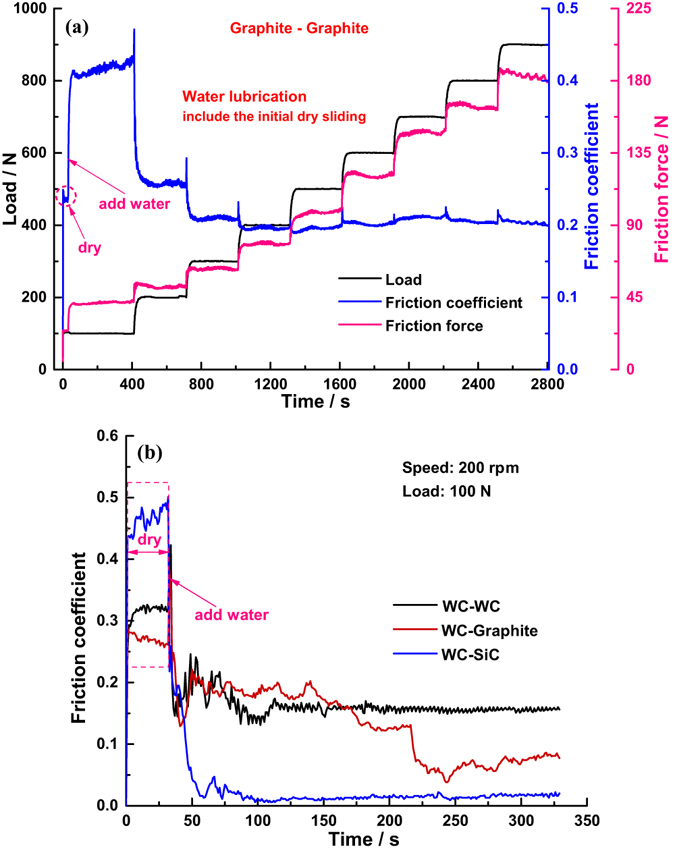
Variation of friction coefficient with running time under different external loads at a rate of rotation of 200 rpm, corresponding to 0.5 m/s, from initial dry sliding to the addition of water: (**a**) of phenolic resin-impregnated graphite sliding against itself, and (**b**) of cemented carbide (WC) sliding against itself, WC sliding against graphite and WC sliding against SiC.

In order to explain the increase of friction coefficient when adding water into the initial dry friction interface under a load of 100 N, comparative experiments were conducted. However, we found that the friction coefficient between SiC-SiC under water conditions, not including the initial dry sliding stage, was indeed lower than that under dry sliding conditions at the same operating conditions (Fig. 5). We thus proposed that bulk water did not enter the initial dry friction interface due to the very poor wettability of the SiC surface with its water contact angle of 86°; there was only small amounts of water in the interface, which formed enormous capillary pressure, causing a strong attraction between asperities, between wear debris, and between asperities and wear debris so that the friction coefficient dramatically increased when keeping the nominal load constant, the influence mechanism schematic of which is shown in Fig. 6(a). To test this assumption, we measured the friction coefficient of phenolic resin-impregnated graphite sliding against itself (i.e. the moving and static rings materials are the same) with a water contact angle of 109°, and also observed the increase when adding water to the initial dry friction interface [Fig. 7(a)]; in contrast, if one or two surfaces were hydrophilic, such as cemented carbide with a water contact angle of 60°, the friction coefficient would decrease after adding water [Fig. 7(b)]. These demonstrated that poor wettability of both surfaces leaded the bulk water not to enter the dry friction interface. Meanwhile, we also performed another experiment, i.e. applying load first and then adding water into the liquid pool until the water level submerged the friction interface [Fig. 5(c)], contrary to the sequence shown in Fig. 5(b). Comparing the two figures, we found that the friction coefficient under a load of 100 N [Fig. 5(c)] was higher than that for the contrary sequence [Fig. 5(b)], resulting from the initial running stage where bulk water did not enter the friction interface thus generating a large friction force; nevertheless, compared with the friction coefficient under the same load of 100 N in Fig. 1, it was lower than that under dry friction conditions, and was far below that measured after the addition of water. The main difference between the two experiments in Figs 1 and 5(c) was that, before adding water, wear debris was present in the former friction interface due to the initial dry sliding shown in Fig. 3(a) and the formation of rougher surfaces shown in Fig. 4 also arising from this, meaning that these two factors played a critical role in small amounts of water forming this enormous capillary pressure.

Moreover, movement of wear debris during the running process generated strong interface adhesion which continuously underwent the process of formation, destruction, and reformation, thereby resulting in the large fluctuation of the friction coefficient, even far exceeding that under dry friction conditions, and causing the slight vibration of the liquid pool. While there was no obvious large fluctuation in the friction coefficient between graphite-graphite when adding water after initial dry sliding [Fig. 7(a)], indicating that the distribution of graphite wear debris is relatively uniform. Besides, due to the hardness of graphite is far below that of SiC, the dimension of graphite debris might be larger than that of SiC under the same load; and Laplace pressure in capillary bridge is proportional to the reciprocal of curvature radius, indicating that the capillary pressure between wear debris and between wear debris and asperities was much smaller than those in SiC. As a result, the change in friction force resulting from the movement of graphite wear debris was relative small.

### Capillary bridges break

As the load increased to 200 N, the friction force decreased abnormally, meaning the slope of the friction force against load curve is negative. Figure 6(b) shows that the increase of external load made the solid contact pressure and area between asperities, between wear debris, and between asperities and wear debris largely increased, leading to the small amounts of water within the three parts to be squeezed out from the capillary bridges to become free water; and the reduction of the number of capillary bridges across the whole interface caused the large total capillary pressure to rapidly decrease, which indicated that equivalent external load was suddenly release, thus causing the micro-separation of the interface a sudden increase which then generated the passages through which bulk water entered the friction interface. When the load further increased to 300 N, the amount of free water in the interface increased, resulting in the capillary pressure tending to zero, and large wear debris with dimensions exceeding the water film thickness being washed away from the interface due to water flow during operation, leading to the fluctuations in measured friction coefficient vanishing and the fluctuation pattern becoming almost identical to that observed under water lubrication not including the initial dry sliding [comparing Fig. 1(a) with Fig. 5(b)]. Thus both factors caused the friction force to undergo further decreases. Actually the specific value of the friction coefficient of graphite sliding against itself under a load of 200 N was roughly equivalent to that for SiC, but the extent of the influence of capillary pressure under a load of 100 N in the graphite-graphite interface was smaller than that for SiC, so the abnormal decrease of friction force with increasing load did not appear in graphite [Fig. 7(a)], or in the experiment applying load first, and then adding water [Fig. 5(c)].

### Adhesion effect

Once the load exceeded 700 N, the friction force sharply increased immediately, even causing the rotation speed of the tribometer to decrease enforcedly; when the load reached 800 N, the rotator stop completely. The same phenomenon and the same critical load zone were found in the two experiments under water lubrication (Fig. 5(b,c)). It is well known that the higher the external load, the smaller the water film thickness, so when the load reaches a certain value, exceeding the maximum carrying capacity of the water film, the water in the interface will be squeezed out, leading to solid-solid contact area and contact pressure increases so that the friction force must increase. As mentioned above, small amounts of water caused enormous capillary force that was equivalent to an increase of external load, whether it is reason enough to make the rotator stop is a moot point. However, it was that, under dry friction conditions, water was not present in the dry interface, during the loading process from 500 N to 600 N, the rotator was also forced to reduce speed until eventually stopping completely [Fig. 5(a)], as caused by the solid contact pressure exceeding the maximum load capacity of the tribometer motor. The difference was that the critical load zone under dry conditions was lower than that under water condition, which was due to the water film in the interface possessing a certain carrying capacity preventing the two surfaces from making contact within certain limits. By contrast, over the same load range from 100 N to 900 N and in an almost identical lubrication state – mixed lubrication that can be obtained from the friction coefficient according to the classical Stribeck curve, this high solid contact pressure did not appear in the graphite-graphite friction interface [Fig. 7(a)], as well as in the WC-WC, WC-SiC, and WC-Graphite friction interfaces; along with JKR theory, we can conclude that the adhesion force between SiC-SiC is much stronger than that between other materials, which is the major component of the solid contact pressure. So, strong adhesion force between SiC-SiC in a certain load range is the real reason why the friction force sharply increases, as shown in Fig. 6(c). This may also be the primary reason why the friction components ofSiC sliding against itself are nearly never applied to low-pair contact such as mechanical seals and thrust bearings by the world’s three leading nuclear main pump supply corporations (*i.e*., EMD in the USA, KSB in Germany, and Framatome in France) even if severe wear of water lubrication thrust bearings has become the bottleneck restricting the working efficiency and reliability of shield coolant pumps in third-generation nuclear technology.

In conclusion, we found that there were some unexpected friction behaviours arising in the SiC-SiC friction interface from initial dry sliding to the addition of water. Due to the poor wettability of the SiC surface, bulk water could not enter the initial dry friction interface, and only small amounts of water was present in the interface, forming enormous capillary pressure and causing a strong attraction between asperities, between wear debris, and between asperities and wear debris so that the friction coefficient under water lubrication was far greater than that under dry friction conditions. With increasing external load, the solid contact pressure and area largely increased, resulting in these small amounts of water being squeezed out from the capillary bridges to become free water. The capillary pressure rapidly decreased, thus causing the micro-separation of the interface to suddenly increase, thus generating the passages through which bulk water could enter the friction interface. So, the abnormal phenomenon by which the friction force decreased with increasing external load occured in the SiC-SiC friction interface. As the load further increased, the friction force increased in a quasi-linear fashion, in accordance with Amontons’ law. However, once the load reached the critical load zone, the water film was squeezed out from the interface; along with the strong adhesion force between SiC-SiC, the equivalent load on the interface was far greater that the maximum load capacity of the tribometer motor, making both the friction coefficient and friction force increases more than five-fold, thus bringing the rotator to an enforced stop. These findings are of great significance for perfecting classical tribology theory.

## Materials and Methods

### Materials

The materials used in the research were SiC rings, which were fabricated by a pressureless sintering process and were composed of high-density granular α-SiC without free carbon graphite and silicon. The elastic modulus is 374 GPa, Vickers hardness 21.4 GPa, and thermal conductivity 851.4 μJ/(mg∙K). The SiC ring porosity is less than 2.5%, as measured by mercury intrusion porosimetry with a Micromeritics Autopore IV 9500. Moreover, to reduce the influence of form tolerance and position tolerance on the experimental results, the parallelism between the friction surface and support surface for both upper and lower rings are required to be less than 0.01 mm, which was ensured by using a face-grinding machine; both rings were ground to a flatness of less than 1 μm and surface roughness of less than 0.1 μm using a diamond slurry on a brass grading plate^19^.

### Tribological tests

**Figure 8:**
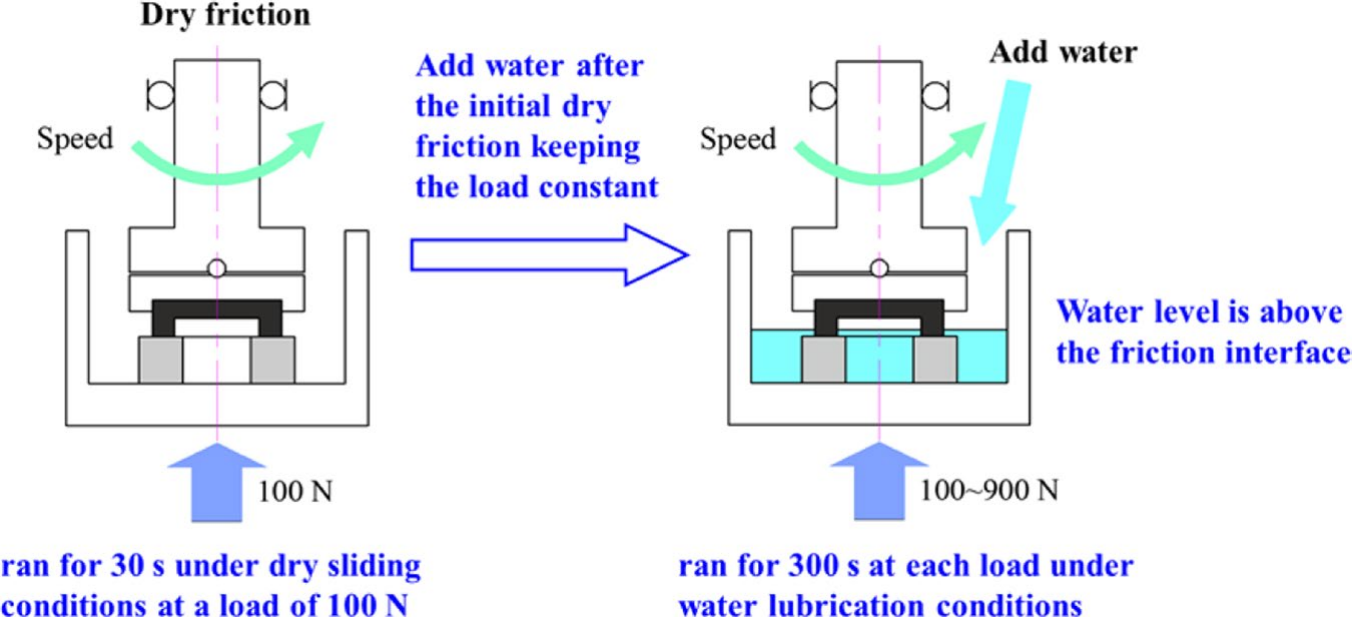
Experiment procedures: ran for 30 s under dry sliding conditions at a load of 100 N, then adding water into the liquid pool until the water level is above the friction interface, and continued running for 300 s; subsequently, a load of 200–900 N was applied by the pneumatic load system and the running time at each load was also 300 s.

The experiments were performed under ambient conditions, room temperature of 25 °C and the relative humidity of 30%, using a ring-on-ring model, Plint TE92, tribometer [inset, Fig. 1(a)]. The upper rotating SiC ring was attached to an upper holder containing a ball joint that could automatically self-align to avoid eccentric contact; while the lower stationary SiC ring was attached to the inner holder of the liquid pool that is fixed on a thrust bearing. The experimental rate of rotation of 200 rpm was selected to provide a linear velocity of 0.5 m/s, corresponding to the opening speed during start-up of water lubrication thrust bearings in some typical shield pumps. The whole experimental procedure may be summarised as follows: in the initial stage, the SiC-SiC friction component ran for 30 s under dry sliding conditions at a load of 100 N; then adding water into the liquid pool until the water level is above the friction interface to submerge it, and continued running for 300 s under constant operating conditions; subsequently, a load of 200–900 N was applied by the pneumatic load system to investigate the effect of external load on the friction behaviour under water lubrication. The running time at each load was also 300 s. The above experiment procedures are shown in Fig. 8.

### Characterization

A white light interferometer (Nexview, Zygo, USA) was used to assess the surface micro-asperities and calculate surface roughness before and after the initial dry sliding stage. An optical microscope was used to observe the wear debris on the SiC surface after the initial dry sliding stage.

## References

[1] Bhushan B (2002). Introduction to Tribology.

[2] Wen S, Huang P (2012). Principles of Tribology.

[3] Fall A (2014). Sliding friction on wet and dry sand. Phys. Rev. Lett..

[4] Scheel M (2008). Morphological clues to wet granular pile stability. Nat. Mater..

[5] Siavoshi S, Orpe AV, Kudrolli A (2006). Friction of a slider on a granular layer: nonmonotonic thickness dependence and effect of boundary condition. Phys. Rev. E..

[6] Persson N (2000). Sliding Friction: Physical Principles and Applications.

[7] Géminard J–C, Losert W, Gollub JP (1999). Frictional mechanics of wet granular material. Phys. Rev. E.

[8] Berman D (2015). Macroscale superlubricity enabled by graphene nanoscroll formation. Science.

[9] Erdemir A, Donnet C (2006). Tribology of diamond-like carbon films: recent progress and future prospects. J. Phys. D Appl. Phys..

[10] Jiang JR, Zhang S, Arnell RD (2003). The effect of relative humidity on wear of a diamond-like carbon coating. Surf. Coat. Tech..

[11] Andersson J, Erck RA, Erdemir A (2003). Frictional behaviour of diamondlike carbon films in vacuum and under varying water vapour pressure. Surf. Coat. Tech..

[12] Kobayashi M, Takahara A (2010). Tribological properties of hydrophilic polymer brushes under wet conditions. Chem. Rec..

[13] Yang J (2015). Sliding friction and wear behaviors of plasma sprayed alumium-bronze coating in artificial seawater. Surf. Interface Anal..

[14] Deng Z (2012). Adhesion-dependent negative friction coefficient on chemically modified graphite at the nanoscale. Nat. Mater..

[15] Thorman E (2013). Negative friction coefficients. Nat. Mater..

[16] Bodvik R, Thormann E, Karlson L, Claesson PM (2011). Temperature responsive surface layers of modified celluloses. Phys. Chem. Chem. Phys..

[17] Trinh LTT (2010). Temperature-induced adsorption and optical properties of an amphiphilic diblock copolymer adsorbed onto flat and curved silver surfaces. J. Colloid Interf. Sci..

[18] Dedinaite A (2010). Friction in aqueous media tuned by temperature-responsive polymer layers. Soft Matter..

[19] Guo F, Tian Y, Liu Y, Wang Y (2016). Ultralow friction between cemented carbide and graphite in water using three-step ring-on-ring friction test. Wear.

